# Disruption of columnar and laminar cognitive processing in primate prefrontal cortex following cocaine exposure

**DOI:** 10.3389/fnsys.2015.00079

**Published:** 2015-05-29

**Authors:** Ioan Opris, Greg A. Gerhardt, Robert E. Hampson, Sam A. Deadwyler

**Affiliations:** ^1^Department of Physiology and Pharmacology, Wake Forest University School of MedicineWinston-Salem, NC, USA; ^2^Department of Anatomy and Neurobiology, University of KentuckyKentucky, KY, USA

**Keywords:** prefrontal cortex, columnar processing, pyramidal cell, interneuron, executive control, nonhuman primates, cocaine, target selection

## Abstract

Prefrontal cortical activity in primate brain plays a critical role in cognitive processes involving working memory and the executive control of behavior. Groups of prefrontal cortical neurons within specified cortical layers along cortical minicolumns differentially generate inter- and intra-laminar firing to process relevant information for goal oriented behavior. However, it is not yet understood how cocaine modulates such differential firing in prefrontal cortical layers. Rhesus macaque nonhuman primates (NHPs) were trained in a visual delayed match-to-sample (DMS) task while the activity of prefrontal cortical neurons (areas 46, 8 and 6) was recorded simultaneously with a custom multielectrode array in cell layers 2/3 and 5. Animals were reinforced with juice for correct responses. The first half of the recording session (control) was conducted following saline injection and in the second half of the same session cocaine was administered. Prefrontal neuron activity with respect to inter- and intra-laminar firing in layers 2/3 and 5 was assessed in the DMS task before and after the injection of cocaine. Results showed that firing rates of both pyramidal cells and interneurons increased on Match phase presentation and the Match Response (MR) in both control and cocaine halves of the session. Differential firing under cocaine vs. control in the Match phase was increased for interneurons but decreased for pyramidal cells. In addition, functional’ interactions between prefrontal pyramidal cells in layer 2/3 and 5 decreased while intra-laminar cross-correlations in both layers increased. These neural recordings demonstrate that prefrontal neurons differentially encode and process information within and between cortical cell layers via cortical columns which is disrupted in a differential manner by cocaine: administration.

## Introduction

Cognitive deficits related to drug addiction, aging, attention deficit hyperactivity disorder (ADHD), schizophrenia and autism are characterized by the inability to select correct behavioral responses for the appropriate environmental or task related circumstances (Shallice and Burgess, 1991; Duncan et al., 1997; Buxhoeveden and Casanova, 2002; Buxhoeveden et al., 2006; Beveridge et al., 2008; Brennan and Arnsten, 2008; Dobbs, 2010; Wang et al., 2011). A common feature of such cognitive deficits in primate brain is the disruption of neural activity in prefrontal cortex (PFC), namely the precise laminar/columnar organization of neural firing that coordinates cognition and behavior (Mountcastle, 1997; Rao et al., 1999; Beveridge et al., 2008; Weiler et al., 2008; Opris et al., 2012a). Such cognitive processing can be disrupted by extended exposure to commonly abused drugs, such as cocaine (Opris et al., 2009, 2012a; Deadwyler, 2010). It is known that prefrontal cortical activity in the primate brain plays a critical role in cognitive processes such as working memory and the executive control of behavior. Groups of prefrontal neurons within specified cortical minicolumns use inter-laminar regular spiking and intra-laminar fast spiking to process relevant information for goal oriented behavior (Kritzer and Goldman-Rakic, 1995; Constantinidis and Goldman-Rakic, 2002; Opris et al., 2011, 2012a,b, 2013, 2014; Opris, 2013; Opris and Casanova, 2014).

However, what is not known is how a drug like cocaine modulates such micro-anatomical columnar and intra-laminar spiking in prefrontal cortex. Rhesus macaque nonhuman primates (NHPs) were trained in a visual delayed match-to-sample (DMS) task while the activity of prefrontal cortical neurons (areas 46, 8 and 6) was recorded simultaneously with a custom multielectrode array in cell layers 2/3 and 5. In order to test specificity of laminar-columnar firing for correct performance in the task, a known cognitive impairing drug, cocaine, was administered midway through each session which *in*duced a change in task-related mini-columnar processing in the same manner as when errors occurred under normal, nondrug, conditions (Opris et al., 2012a). Results revealed that differential firing in the cocaine vs. control halves of the session increased for interneurons and decreased for pyramidal cells during the Match phase of the task. Interestingly, columnar interactions between pyramidal cells in layer 2/3 and 5 decreased during task performance with cocaine, while intra-laminar cross-correlations between cells with higher firing rates increased. The results demonstrate that prefrontal cortical neurons differentially encode and process information within and between layers in the cortical columns in a manner sensitive to alterations provoked by the addicting drug, cocaine.

## Methods

All animal procedures were reviewed and approved by the Institutional Animal Care and Use Committee of Wake Forest University, in accordance with U.S. Department of Agriculture, International Association for the Assessment and Accreditation of Laboratory Animal Care, and National Institutes of Health guidelines.

### Rule-Dependent Delayed-Match-to-Sample (DMS) Task

**Figure 1:**
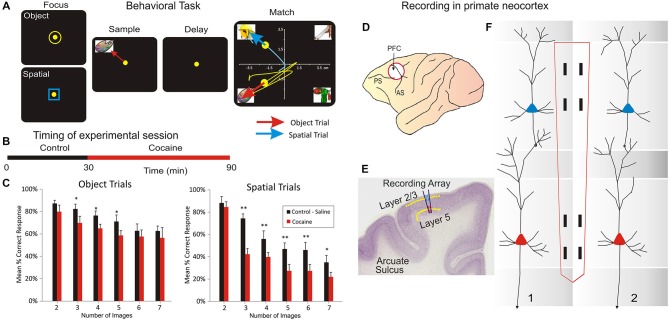
**Inter-laminar neuron recording in Nonhuman primates (NHPs) performing a visual delayed-match-to-sample (DMS) task which required movement of a cursor into images projected onto a video screen. (A)** Behavioral paradigm showing the four phases of the DMS task: (1) Focus Stage: presentation of a circle or square image to initiate with cursor placement either an Object or a Spatial type of trial followed by, (2) presentation of the trial “Sample” image, followed by cursor placement in the image as the “Sample Response” which initiated (3) a variable “Delay” period with the screen blank for 5–60 s which on termination initiated (4) the “Match” phase in which the Sample image was presented simultaneously with a random number of 1–6 other Non-match (distracter) images on the same screen. Cursor movement for a duration of ≥0.5 s into (a) the Sample image for correct performance on Object trials, or (b) the same spatial position on the screen that the Sample image occupied, for correct performance on Spatial trials, produced a juice reward via a sipper tube mounted next to the animal’s mouth. Placement of the cursor into a non-match “distracter” image for ≥0.5 s constituted an error and caused the trial to terminate and the screen to blank without reward delivery. The Inter-trial interval (ITI) was 10.0 s for all trials. **(B)** DMS session timeline shown for combined Control (saline) and Drug (cocaine) administration phases of the same trial. **(C)** Mean percent correct performance on Spatial and Object trials averaged across all animals (*n* = 4) for trials with different numbers of distracter images (1–6) in the Match phase of the DMS task. **(D)** NHP brain profile showing the placements of recording Multielectrode arrays (MEAs) in the Prefrontal Cortex. **(E)** Illustration of coronal section in NHP frontal cortex showing relative placement location of MEA probes in supra-granular layer 2/3 and infra-granular layer 5 shown in **(F). (F)** Dimensionally relevant illustration of the conformal MEA positioned for simultaneous recording from neurons in both layers via adjacent minicolumns (1 and 2), consisting of a “pair” of cells recorded with one in L2/3 and the other in L5 PFC for each minicolumn. Reprinted with permission from Opris et al. (2012a).

The NHPs utilized as subjects in this study (*n* = 4 male, weight 5–8 kg) were trained for at least 1 year to perform a well characterized, custom-designed visual delayed-match-to-sample (DMS) task (Hampson et al., 2010, 2011; Opris et al., 2005, 2011; Hampson et al., 2012a,b) which is shown in Figure 1A. During testing in DMS task, animals were seated in a primate chair with a shelf-counter in front of a display screen (Figure 1A) such that right arm position on the counter top was tracked via a UV-fluorescent reflector affixed to the back of the wrist, which was illuminated with a 15 W UV lamp detected by a small LCD camera positioned 30 cm above the hand. Hand position and movement was digitized and displayed as a bright yellow cursor on the display screen. Trials were initiated by the animal placing the cursor inside a “Start” shape (3” diameter yellow ring or blue square) in the center of the screen (Figure 1A). Then, a trial-unique “Sample” clip-art image is displayed randomly at one of 8 different spatial positions on the screen for 2, 0 s (“Sample Phase”). NHPs were required to place the cursor in the Sample image (Sample Response) to initiate the Delay phase of the task in which the screen was blanked for a random duration of 1–60 s, on any given trial.

Based on the “Start” shape (yellow ring or blue square) the animal was instructed to remember the feature/object (yellow ring) or the spatial location (blue square) of the Sample image. Alter the conclusion of Delay interval is initiated the presentation of the Match phase of the task, in which a screen display of 2–7 trial unique clip-art images, including the Sample image, were displayed simultaneously at separate, randomly selected spatial locations. Placing the cursor into the Sample image during the Match phase constituted the correct “Match Response” which produced a drop of juice delivered via a sipper tube located near the animal’s mouth and blanked the screen. Placement of the cursor into one of the non-match (distracter) images constituted a non-match (error) response and caused the screen to blank without reward delivery and initiated the 10 s inter-trial interval (ITI). All images (sample and distracter) were unique for each trial in sessions of 100–150 trials and were chosen from a 5000 image buffer which was updated with new images every month. All NHP subjects were trained to overall performance levels of 70–75% correct on the above described DMS task parameters. The DMS task was split (Figure 1B) on two parts: (a) the control phase (in which the animal was injected with saline after 30 min); and (b) cocaine phase, following IV injection of cocaine. Behavioral performance in the DMS task (Figure 1A) is shown in Figure 1C.

### Surgery

Animals were surgically prepared with cylinders for attachment of a microelectrode manipulator over the specified brain regions of interest. During surgery animals were anesthetized with ketamine (10 mg/kg), then intubated and maintained with isoflurane (1–2% in oxygen 6 l/min). Recording cylinders (Crist Instruments, Hagerstown, MD) were positioned over 20 mm diameter craniotomies for electrode access (Hampson et al., 2011) to stereotaxic coordinates of the Frontal Cortex (25 mm anterior relative to interaural line and 12 mm lateral to midline/vertex) in the caudal region of the Principal Sulcus, the dorsal limb of Arcuate Sulcus in area 8 and the dorsal part of premotor area 6 (Figure 1D), areas previously shown by PET imaging to become activated during task performance (Figure 1C; Hampson et al., 2011). Two titanium posts were secured to the skull for head restraint with titanium steel screws embedded in bone cement. Vascular access ports (Norfolk Medical Products, Skokie, IL) for drug infusions were implanted subcutaneously in the mid-scapular region, for a femoral incision, and flushed daily with 5 ml heparinized saline needed for IV drug administration.

### Electrophysiological Recording

**Figure 2:**
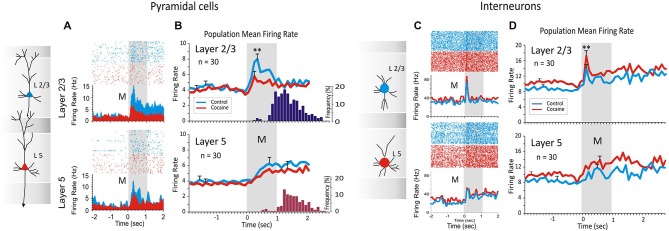
**Inter-laminar activity recorded from prefrontal cortical neurons (regular spiking pyramidal cells) (A) and (B), and fast spiking interneurons (C) and (D), during DMS task performance**. Cocaine administration (0.30, 0.40, 0.60 and 0.90 mg/kg IV) decreased inter-laminar pyramidal cell firing and conversely increased interneuron laminar firing during DMS performance. **(A)** Raster-PEHs of layers 2/3 and 5 inter-laminar pyramidal cell pair firing at the time of the Match Response (MR) during the Match phase (a) in the initial control (saline IV) half of the session (blue); and (b) following cocaine administration (red) midway through the session (Figure 1B). **(B)** Average PEHs for trials in the control (upper) vs. cocaine half of sessions (lower) summed over all inter-laminar layer 2/3 (blue) and layer 5 (red) PFC cell pairs (*n* = 30) in sessions in which cocaine was administered, as shown in Figure 1B. Dark blue and red histograms on the right show average distribution of MR latencies relative to Match phase onset (M, 0.0 s) for control and cocaine trials respectively, within the same sessions. **(C)** Raster-PEHs show firing of layer 2/3 and 5 interneurons in the Match phase during the control (saline) half of the session (blue) vs. cocaine administration midway through the session (red). **(D)** Average PEHs for control (upper) vs. cocaine trials (lower) summed over all fast spiking interneurons (*n* = 30) in layer 2/3 (blue) and layer 5 (red) recorded in the same sessions. (**p < 0.01, ANOVA). Panels **(A)** and **(B)** are adapted with permission from Opris et al. (2012a).

Electrophysiological procedures and analysis utilized the MAP Spike Sorter by Plexon, Inc (Dallas, TX) for 64 channel simultaneous recordings. Customized conformal designed ceramic multielectrode arrays (MEAs) were manufactured in collaboration with Dr. Greg Gerhardt (Center for Microelectrode Technology—CenMet, Lexington, KY) at the University of Kentucky (50). MEAs consisted of eight etched platinum pads (Figures 1E,F) for recording multiple single neuron activity (Hampson et al., 2004, 2011) from which single extracellular action potentials (Figures 2A,C) were isolated and analyzed with respect to firing on specific recording pads during different events within DMS trials (Figure 1). The model W3 configuration probe with the recording pads are vertically separated by 1350 um (Figure 1F) was specially designed to conform to the columnar anatomy of the PFC such that the top 4 recording pads recorded activity from neurons in the supra-granular layer 2/3 while the lower set of four pads simultaneously recorded neuron activity in the infra-granular layer 5 (Figure 2).

### Data Analysis

**Figure 3:**
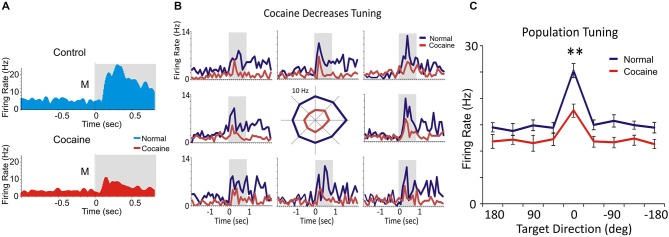
**Spatial tuning. (A)** Match phase response firing of a layer 2/3 PFC cell firing during the saline control (blue) and cocaine (red) administration segments of daily DMS sessions. **(B)** Multigram spatial tuning plot (reference here) shown as multiple PEHs (multigram) for spatial tuning of target selection (diagram in center) for two PFC layer 2/3 cell pairs during control (blue) and cocaine (red) halves of the daily session. The spatial tuning plot in the middle displays Match phase mean firing rates (shaded areas in PEHs) along each radial axis corresponding to movement of the cursor into each of the 8 screen image positions from the screen center summed over all trials in a single session. The spatial “bias” for minicolumn firing (layers 2/3 and 5 cells) is indicated by an increased firing rate for target selection in one position (i.e., left 180° position) vs. all others during the session. **(C)** The comparison of spatial tuning across all PFC neurons indicates decreased spatial preference for minicolumnar firing (layers 2/3 and 5 cells) after cocaine administration in the same session. **p < 0.001, ANOVA.

Task performance was determined for each animal (*n* = 4) as % of correct trials within and across sessions related to simultaneous recordings of MEA conformal single neuron firings on individual trials during Match phase image selection in the task (Hampson et al., 2011). Cell types were identified as function of changes (*z* > 3.09, *p* < 0.001) in firing rate (see below) on single trials in perievent histograms (PEHs) derived for intervals of ±2.0 s relative to the time of Match screen presentation (0.0 s) that signaled onset of the Match phase of the task (Figures 1–3). Task-related neural activity was classified according to locations on the conformal MEA positioned specifically in cortical layers 2/3 and 5 (Figure 1F) prior to each DMS session. To account for neuronal responses in terms of columnar microcircuit organization, PFC neurons recorded on the MEAs were characterized by (1) layer specific firing in terms of simultaneous cell activity on both vertical pads (Figure 1F) during electrode positioning; and (2) whether the same cell pair firing was specifically modulated during the Match phase of the DMS task (Hampson et al., 2011; Opris et al., 2011). It is beyond the scope of this paper to analyze neural activity based on the “Start” ring/square shape (spatial vs. object), that was previously reported in Opris et al. (2013). Standard scores, *Z* = [peak − baseline firing rate]/SD baseline firing rate, were calculated for individual cell firing on each DMS task event. Neurons were only included in the analysis if their firing rates were significantly elevated from that in the Pre-Match presentation (−2.0–0.0 s) baseline period (*Z*-scores, ANOVA *F* test *p* < 0.01).

This study compares the rapid spiking of interneurons across prefrontal cortical layers with the previous published regular spiking activity in pyramidal cells under cocaine vs. control (Opris et al., 2012a). Cell type classification in putative pyramidal and interneurons (Constantinidis and Goldman-Rakic, 2002) is based on the general idea that interneurons have higher baseline firing (>10 Hz) than the pyramidal cells (<10 Hz). However, this study does not target firing of specific type of interneurons (such as chandelier, basket, double bouquet or Martinotti cells). Statistical Analyses for inter-laminar differences in firing for cells in layers 2/3 vs. 5 during the Match Phase were assessed first.

**Figure 4:**
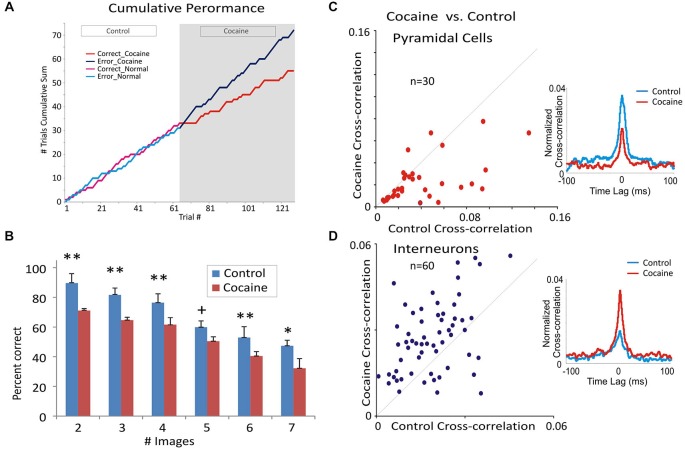
**Cocaine administration effect on behavioral and neural correlates of DMS performance. (A)** Single session example of the change in cumulative distribution of correct and error trials during control (saline) vs. cocaine segments of the session. Cocaine (0.40 mg/kg) was administered after trial #62 of the session, which reset the cumulative trial plot (trial #62) for the remaining 61 trials in the cocaine half of the session. It is clear that cocaine produced a marked change in the cumulative number of correct (red) and error (blue) trials across the cocaine half of the session (last 61 trials) compared to the prior saline half of the session where the cumulative error/correct trial distribution (green and yellow) was similar. **(B)** Mean percent correct performance across all animals (*n* = 4) as a function of number of distracter images (1–6) in the Match phase during control vs. cocaine halves of the same session (*n* = 19). **F_(1,96)_ > 11.22, *p* < 0.001; *F_(1,96)_ = 10.07, *p* < 0.01; ^+^*F*_(1,96)_ = 3.87, *p* < 0.05. **(C,D)** Scatter plots of normalized cross-correlation coefficients from inter-laminar pyramidal cell pairs **(C)** and interneurons **(D)**. Insets show opposite variation of the mean cross-correlation histograms CCHs for the same inter-laminar/interneuron cell pairs and in the neural firing in the two subsets of data. Panels **(A)**, **(B)** and **(C)** are adapted with permission from Opris et al. (2012a).

Differences in cross-correlation were assessed using standardized distributions of coefficients extracted from firing of inter-laminar cell pairs under different conditions related to performance in the Match Phase (Figure 2). Mean cross-correlation histograms (CCHs) were calculated and compared relative to normalized mean coefficients (Opris et al., 2011) for the same populations of cell pairs under different experimental conditions (Figure 2). The correspondence of firing between cells in different layers was tested via cross-correlation histograms (CCHs) that extracted synchronous occurrences of spikes in both layers employing layer 2/3 cell firing to test the synchronous discharge of simultaneously recorded layer 5 cells in 1.0 ms intervals over ±1.0–2.0 s in task related events (Figure 2). CCHs for inter-laminar cell pairs (layer 2/3 and 5) were generated using a “shuffle” shift predictor built into NeuroExplorer,^1^ which computed random cross-correlation levels due to chance. The “shift-predicted” chance correlation factor was subtracted from the true coefficients for CCHs to adjust differences in cell firing rates and frequency of bursting (Opris et al., 2011; Takeuchi et al., 2011). Population (mean) CCHs were computed by averaging CCH coefficients across multiple cell pairs and plotting the mean values (±SEM) in 1.0 ms bins (Figures 4C,D).

### Tuning Plots of Prefrontal Cortical Cell Firing

For each inter-laminar cell pair (layer 2/3 and 5) firing rate on the same trial type (control, cocaine) was plotted with respect to the location of the matching image on the screen, in the Match phase (Figure 3). Directionality was assigned according to the 8 positions on the screen with reference to placement of the cursor providing angular directions corresponding to the location of the match image around the periphery of the screen, yielding the following degree movement directions from center of screen: 0° (directly lateral), 45°, 90°, 135°, 180°, 225°, 270°, 315° and 360° (Rao et al., 1999; Felsen et al., 2002). Mean firing rate commencing at Match phase onset until time of occurrence of the MR (i.e., typically 0.5–1.0 s, Figure 3B) was calculated for the position of the response on each trial and represented in polar coordinates as tuning plots of the average firing rate for each inter-laminar cell pair over all trials in a single session. A directional bias, or “preference”, for a given cell pair was assessed by Rayleigh test (employing circular statistics) comparing response locations with the highest mean firing rates relative to all the other positions responded to during the session (Figure 3B). Average tuning plots (Figure 3C) were constructed by averaging firing rates of cells in each layer for each screen position over all trials across all sessions.

### Assessment of Cortical layer and Minicolumn Activity

The conformal MEA (model W3) probe (Figure 1E) was specially designed such that the two sets of recording pads could record simultaneous activity from neurons separated by 1300 μm, which given its orientation of insertion into PFC (dorsal premotor gyrus in area 6, stereotactic coordinates AP:25 and ML:12) constituted columnar firing of cells in infra-granular layer 5 and supra-granular layer 2/3 (Hampson et al., 2004; Hansen and Dragoi, 2011; Opris et al., 2011; Takeuchi et al., 2011) as shown in Figure 1F. Misplacement of the probe due to different angular penetration relative to inter-layer columnar orientation in PFC was directly signaled by the absence of simultaneous cell recordings on both sets of pads separated vertically by 1300 μm. The MEA (Hampson et al., 2004; Opris et al., 2011) employed here allowed recording of PFC columnar activity in two dimensions rather than one (Hansen and Dragoi, 2011; Mo et al., 2011; Takeuchi et al., 2011) since, with proper vertical alignment (<5.0°), activity from two different but adjacent minicolumns could be detected and validated as shown in (Opris et al., 2012a,b).

### Cocaine Administration

Animals were trained to perform the task with IV saline injections into the saphenous vein of the left leg through the vascular access port prior to and midway through DMS testing sessions. For conditions in which cocaine was administered, midsession saline injections were replaced with IV injection of cocaine (0.30, 0.40, 0.60 and 0.90 mg/kg IV), via the same route (Opris et al., 2009, 2012a; Hampson et al., 2011).

## Results

Four NHPs performed the delayed-match-to-sample (DMS) task (Opris et al., 2009, 2012a; Hampson et al., 2011) by selecting the same video image presented on-screen in the prior Sample phase from a set of 2–7 images in the subsequent Match Phase to obtain a juice reward after an intervening Delay of 1–60 s (Figure 1A). Hand tracking movements of a cursor on the screen were made in the Match phase to select the correct (Sample) image which varied with respect to image-type and screen position on each trial. Key variables in the task were (a) number of distracter images (2–7) presented on the screen in the Match phase, (b) duration of the intervening delay (1–60 s) and (c) the random placement of the Sample (target) image in 1 of 7 spatial positions on the screen in the Match phase that differed from the position in the Sample phase. Previous work with the same DMS task validated the important aspects such as attention, short-term memory and response latency, together with influences of decision variables, like cognitive workload and reward expectancy (Porrino et al., 2005; Deadwyler et al., 2007; Opris et al., 2011, 2012a,b, 2013). However, recent analyses have shown that animals executed a “decision process” in the Match phase of the task (Figure 1A) involving target selection, described here in relation to neuron firing in PFC (Opris et al., 2012a,b, 2013). For these experiments we examined the PFC firing from four NHPs in: (a) columnar inter-laminar pair-wise pyramidal cells (*n* = 60 cells) and (b) intra-laminar interneurons (*n* = 60 cells).

### Opposite Trends in Prefrontal Cortical Firing Following Cocaine Administration

In prior investigations (Opris et al., 2012a) the pattern of regular firing during the DMS task has been shown for inter-laminar pairs of pyramidal cells in PFC. In that study (Figures 2A,B) the specific influence of (acute intravenous injection of cocaine (0.4 mg/kg) administered midway through the session) was shown to decrease inter-laminar (layers 2/3 and 5) columnar processing of identified cell pairs in PFC (*n* = 30 cell pairs; 10, 8, 6 and 6 pairs recorded in monkeys K, B, E and G, respectively). Figure 2A shows firing in raster/PEHs for an inter-laminar PFC cell pair (layer 2/3 upper, layer 5 lower) recorded in: (1) the first 60 trials of the DMS session (Control), followed by (2) the same assessment of activity in the second (cocaine) half of the same session (120 total trials) in which cocaine was administered (IV) at trial #61 (Figure 2A, cocaine). Administration of cocaine produced a reduction in Match phase firing of layer 2/3 cell (*Z* score = 7.52, *p* < 0.001) but not layer 5 cell (*Z* score = 1.14, *p* > 0.05, n.s.) in the second half of the session compared to firing of the same cell pair in the control, first half, of the session (Figure 2A). The suppressive effect of cocaine on Match phase mean firing rate over all PFC cell pairs (*n* = 30) is shown in Figure 2B as a significant decrease in layer 2/3 cell activity (*F*_(1,958)_ = 13.43, *p* < 0.001) relative to the saline half of the session, but not in layer 5 (*F*_(1,958)_ = 1.48, *p* > 0.05, n.s.).

Figure 2C,D show intra-laminar fast spiking of prefrontal interneurons (Figures 2C,D) recorded in the same supra- and infra-granular layers. Figure 2C shows firing in raster/PEHs for an inter-laminar pair of PFC interneurons (layer 2/3 upper, layer 5 lower), recorded in the first half of a DMS session (Control) followed by activity assessed in the second half after cocaine was administered (IV) at mid-session (Figure 2C, cocaine). Administration of cocaine produced an increase in Match phase firing of layer 2/3 interneurons (*Z* score = 6.36, *p* < 0.001) but not layer 5 cells (*Z* score = 1.74, *p* > 0.05, n.s.) in the second half of the session compared to firing of the same cell pair in the control (saline injection) first half of the session (Figure 2C). The effect of cocaine over all layer 2/3 cell pairs (*n* = 30) on mean firing in the Match phase is shown in Figure 2D as a significant decrease in activity (*F*_(1,958)_ = 7.12, *p* < 0.001) relative to the first (control) half of the same session. However, the mean firing rate of all simultaneously recorded layer 5 cells was not significantly altered during the same sessions (*F*_(1,958)_ = 1.83, *p* > 0.05, n.s.).

### Cocaine Altered Spatial Tuning of Prefrontal Cortical Cell Firing

Figure 3A shows PEHs of firing of a single layer 2/3 prefrontal cell recorded during saline control (blue) and cocaine (red) halves of the same session which exhibited similar but reduced patterns in firing after cocaine injection for the 8 different locations of target presentation on the screen (Figure 3B). This type of multigram display reflected “tuning biases” or higher firing rates in one vs. other screen locations, which were the same for both cells in a mini-columnar pair (Figure 3B, right; 0° direction). Figure 3C shows a direct comparison of overall firing preference in the control vs. cocaine phases clearly indicating a reduced tuning on cocaine vs. control trials for PFC (*n* = 59) cells (*F*_(1,589)_ = 11.93; *p* < 0.001, ANOVA).

### Cocaine Alters Inter-Laminar and Intra-Laminar Correlated Activity

The effects of cocaine on prefrontal cortical columnar processing are reflected by the decrease in DMS task performance. Figure 4A shows, consistent with prior findings, changes in performance in the same sessions (Figure 2A) on a trial-by-trial basis via injection midway through the session. It is clear that as the number of trials progressed (bifurcation) the cummulative distribution of error and correct trials in the first half of the session (Control, trials 1–61) changed after cocaine administration (trial #61) to cumulation of more errors relative to correct trials in the second half of the same session. Figure 4B illustrates the effects of cocaine on task performance with respect to the decrease (*F*_(1,96)_ = 12.33, *p* < 0.001) in percent correct responses as a function of the increase in the number of distracter images (1–6) in the Match phase during target selection (Opris and Bruce, 2005; Opris et al., 2012a). In association with this decrease in DMS performance, mid-session injection of cocaine produced a significant reduction in the cross-correlation of inter-laminar cell pairs (cocaine cross-correlation in Figure 4C) compared to correlations of the same pairs (*n* = 30) in the Control (saline injection) half of the session. The scatter plot of normalized cross-correlation coefficients (Figure 4C) shows that cell pairs with lower correlation coefficients in the Control half of the session exhibited less change (diagonal line) following cocaine injection than cell pairs with higher coefficients (≥0.04) in the Control half of the session (*F*_(1,59)_ = 11.22, *p* < 0.001). Thus, the higher the inter-laminar correlation under normal conditions, the more likely cocaine reduced that correlation in the same cell pair in the second half of the DMS session. More importantly and in contast, Figure 4D shows a significant increase in the mean CCH peak correlation of interneurons *F*_(1,59)_ = 11.22, *p* < 0.001) in L2/3 and L5 averaged over the same cell pairs (*n* = 60) shown in Figure 2D after cocaine administration.

**Figure 5:**
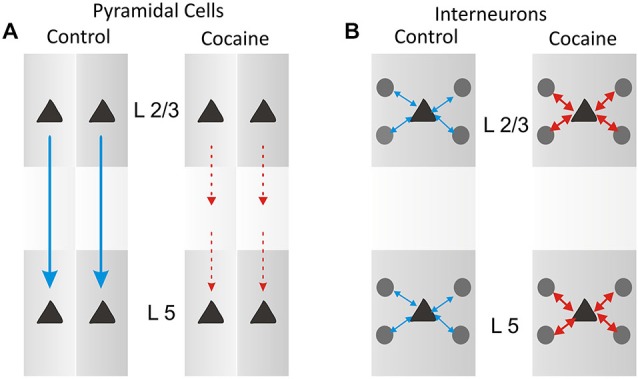
**Schematic diagram for columnar laminar microcircuit interactions illustrating possible underlying basis for the effects of cocaine administration which produced a partial uncoupling (A) of inter-laminar correlated firing between cells in PFC layer 2/3 and layer 5 as shown in Figure 4C, and an increase (B) in firing interneurons in prefrontal cell layers (in Figure 4D)**.

The minicolumnar mechanism of cocaine induced alteration is illustrated in Figure 5A for pyramidal cells as a cocaine-induced decreased columnar transmission between layer 2/3 and 5 cells, which under normal (nondrug) conditions exhibit high levels of firing synchrony as shown in Figure 4C. Consistent with such inter-laminar decrease in correlated firing is the demonstrated increase in the intra-laminar interneuron firing (Figure 5B).

## Discussion

Extensive prior investigation of cognitive processing have shown that activation of PFC is altered by many factors (Opris et al., 2012a,b) including modulation of dopamine influences on task-related PFC cell firing (Bradberry, 2000; Nestler, 2004; Robbins and Arnsten, 2009).

### Alteration in Task-Related Prefrontal Cortical Cell Firing Following Cocaine Administration

Consistent with this notion, PFC neural firing was investigated recently after systemic injections of cocaine in animals performing this DMS task and showed (a) decreased activity in pyramidal cells and (b) increase activity in interneurons, across all trials, which increased the chance of error and reduced performance accuracy (Hampson et al., 2011; Opris et al., 2014). Cocaine blocks the reuptake of dopamine that has been released so dopamine actions are increased in cocaine injected animals. Dopamine modulation of pyramidal (Glutamatergic cells) and interneurons (GABAergic cells) is via release of adrenergic transmitters such as NE (Ramos and Arnsten, 2007) or Serotonin (Celada et al., 2013) to these cells which modifies their ability to react to the excitatory transmitters they normally receive or enhance the release of GABA from interneurons (Fiber and Etgen, 1998). Both actions reduce the firing of pyramidal cells and increases the firing of interneurons due to the differential actions of dopamine receptors (mostly D1) on these cells Ritz et al. (1987).

Although cocaine binds to several sites in the brain, the biochemical receptor mechanism or mechanisms associated with its dependence producing properties are unknown. It is shown here that the potencies of cocaine-like drugs in self-administration studies (Ritz et al., 1987) correlate with their potencies in inhibiting [3H]mazindol binding to the dopamine transporters in the rat striatum, but not with their potencies in binding to a large number of other presynaptic and postsynaptic binding sites. Thus, the cocaine receptor related to substance abuse is proposed to be the one associated with dopamine uptake inhibition.

### Cocaine Induced Disruption of Cortical Columnar Processing

As described in a prior study of the effects of cocaine (Opris et al., 2012a), the reduced correlation of firing between the same inter-laminar cell pairs in the Match phase of the task shown here distinguished a similar cocaine induced reduction in correlated firing for minicolumn cell pairs (Figures 2–4). Cocaine administration therefore enhances dopamine influence on PFC columnar processing by decreasing firing in pyramidal cells and increasing firing in related interneurons. This is shown here by functional interactions between pyramidal prefrontal cells in layers 2/3 and 5 that were decreased while intra-laminar cross-correlation of interneurons increased. These recordings demonstrate that prefrontal cortical neurons differentially encode and process information within critical cortical microcircuits (as shown in the diagram in Figure 5) not only for specific task events, but also with respect to stimulus complexity and reinforcement (Opris et al., 2009, 2012a).

### Differential Dopaminergic Modulation in Prefrontal Cortex

Dopaminergic (DA) receptors D1 and D2 are found on both pyramidal cells and interneurons in prefrontal cortex (Williams and Goldman-Rakic, 1995; Lidow et al., 1998). The differential DA modulation of prefrontal neurons firing under cocaine vs. control conditions was reflected by the increased firing of GABAergic interneurons and the decreased firing of Glutamatergic pyramidal cells (Bradberry, 2000). This is supported by prior evidence in rats showing that DA decreases excitability of layer 5 pyramidal cells (Gulledge and Jaffe, 1998). Also, cells in prefrontal layer 5 bear the majority of mRNAs encoding of all five subtypes of DA receptors (Lidow et al., 1998). In addition, the same opposite trends in functional interactions following cocaine administration are shown in the decreased inter-laminar cross-correlations between pyramidal cell pairs in layer 2/3 and 5, contrasted by the increase in intra-laminar cross-correlations of interneurons in the same layers.

These neural recordings demonstrate that prefrontal neurons differentially encode and process information within and between cortical cell layers via cortical columns which are disrupted in a differential manner by cocaine administration. Such cocaine driven disruption may be explained by a selective reduction of the excitatory synaptic inputs to pyramidal neurons by a selective D1 receptor (Bourne, 2001; Urban et al., 2002; Rebec and Sun, 2005; Stuber et al., 2005; Volkow et al., 2005; Tomasi et al., 2010; Porrino et al., 2013; Santos et al., 2014) with inverted-U dose curve/actions (Vijayraghavan et al., 2007). This view is in agreement to recent studies showing that prefrontal dopamine D1 receptors control visual signals during attention and modulation of memory fields in working memory (Williams and Goldman-Rakic, 1995; Vijayraghavan et al., 2007; Noudoost and Moore, 2011).

## Conclusion

Our unique results provide new insight into the critical role of cortical microcircuits involved in cognition that are altered by drug addiction. These findings clearly demonstrate the susceptibility of cortical information processing to agents that provoke addiction and therefore provide a basis for reversal of these cognitive effects with other agents that (a) promote interlaminar transmission or (b) reduce interneuron firing provoked by an addictive drug like cocaine.

## Conflict of Interest Statement

The authors declare that the research was conducted in the absence of any commercial or financial relationships that could be construed as a potential conflict of interest.

## Footnotes

^1^http://www.neuroexplorer.com/
